# Supplementary material for: Leveraging Grief: Involving Bereaved Parents in Pediatric Palliative Oncology Program Planning and Development
Source: Children (Basel). 2021 Jun 3;8(6):472. doi: 10.3390/children8060472 (PMC8226717; doi:10.3390/children8060472)
Supplement: Supplementary file 1 [file children-08-00472-s001.zip › children-1209098-supplementary.pdf]

Supplemental File S1:

Bereaved Parent Focus Group Guide

Wendy Avery, a founding member of the Quality of Life Steering Committee, developed a focus group guide for use at other institutions that are interested in garnering support for pediatric palliative care interventions. The guide is presented here for hospital and academic use.

How to Use This Guide:

This guide is meant to be used by those who are facilitating a bereaved parent focus group and NOT by the actual participants. It includes 8 topics with starter questions for discussion:

- Overall Quality of Life
- Symptom Control
- Relationship-Based Care
- Useful and Reliable Information
- Making Difficult Decisions
- Care Coordination
- Care During Progressive and Incurable Disease
- Bereavement Support

These topics are NOT meant to be used in defined order but in order of importance according to the individual needs of your institution and what information you would like to gather. (Ex: If you are hoping to gain understanding of the parent's grief journey since the death of their child, start with the topic "Bereavement Support". If you would like to better understand how parents made the transition to palliative care, start with the topic "Care During Progressive and Incurable Disease".)

First Steps:

- Plan potential dates, times, and location of your focus group meetings. You may want to plan on meeting for 2 hours up to 3 separate times and adjust as necessary. The location should be in a place that is private and comfortable for those participating.

• Identify and contact a group of 12-15 bereaved parents willing to participate in your focus group. Be clear about the purpose of this group when asking them to participate.

• Identify 2-3 staff persons to be in attendance. One will act as facilitator. Two will be notetakers.

First Meeting:

• Be sure that the meeting space is comfortable and ready for the participating parents. Provide water, coffee, and possibly a small snack. Have notebooks and pens available for everyone in attendance.

• Greet parents and start the meeting with introductions followed by a clear description of the purpose of the focus group.

• The facilitator will begin by asking questions on the topic that is the focus of the group.

• Then the most important part of the meeting: LISTEN! And take notes on what you are hearing.

• Adjust your questions depending on what you are hearing from your parents. Often, their responses will generate additional questions that are not included in this guide. Be flexible!

• Close your meeting by asking the parent participants how the experience was for them. Did this cause distress? Did they feel a sense of purpose?

• Confirm the date/time for your next focus group.

• Be aware of parents who can communicate effectively and whose answers are presented in a constructive way instead of simply as a complaint. Those are the parents who you may ask to continue to work with you on these efforts after the focus group is completed and you are ready to move forward.

Bereaved Parent Focus Group Questions:

Overall Quality of Life:

- Do you feel that the care your child received throughout their illness/treatment was individualized to the needs of your child? Your family?

    - If yes, give examples.
    - If no, what could have been done to better meet your needs?
- Do you feel the emotional and spiritual needs of your child and your family were addressed during the time of your child's illness/treatment?
    - If yes, give examples.
    - If no, what could have been done to better meet these needs?
- Were your families personal or cultural values taken into consideration during this time?
    - If so, in what ways?
    - If not, what could have been done differently?

Symptom Control:

- Do you feel that your child suffered or was in pain at any point throughout their illness/treatment?
    - If yes, what was that like for you?

Was your child's primary care team or hospice team adequately responsive to your child's suffering? Give examples.

Do you feel that anything could have been done to provide better pain and symptom control to your child? If so, what might have helped?

Relationship-Based Care:

- How would you characterize the relationship between you and your child's care team?
- Do you feel as if you were on the same "team" as the members of your child's care team?
- Do you think that your child's and your family's values and wishes were honored throughout your relationship?
- Did you feel that your child's care team advocated for the comfort and quality of life of your child?
- Did you ever have a negative relationship with anyone caring for your child? If yes, what was that like?
- What were examples of compassionate care from your care team that have stuck with you?

Useful and Reliable Information:

- Would you say that you received honest and transparent information about the health of your child that you could easily understand? Give examples.
- During your child's illness/treatment, did you have access to reliable information so that you could learn more and be better informed while making decisions about the care and treatment of your child?
- Do you feel that your care team was honest and transparent when it came to discussing the possibility that your child may die? If not, do you wish they would have been?
- Do you feel that you were as prepared as possible for the death of your child? If yes, how were you prepared? If no, how could your care team have helped you to be better prepared?

Making Difficult Decisions:

- Did you feel supported by your child's care team when making difficult decisions about the care or treatment of your child?
- Did you ever make a decision about your child's care/treatment that was not what your care team recommended? What was that experience like?
- Are there any decisions made about the care/treatment of your child that you regret? What has that been like for you?
- Is there anything your care team could have done that might have helped prevent that regret?

Care Coordination:

- During your child's illness/treatment did you feel that everyone on your child's care team was on the same page?
- Did you ever get conflicting or confusing information from different members of your child's care team?
    - What was that like?
- Did you feel that you could speak up and ask questions to clarify confusing information?
    - Why or why not?

Care During Progressive and Incurable Disease:

- Were you able to be in the location that you and your child wanted to be when he/she died?
    - If no, why not?
    - If yes, how were you able to make that happen?
- If your child died in the hospital, what was the inpatient room like? For your child? For you? For your family?
- What would have made the room more comfortable during that most difficult time?
- If your child died at home, did you feel supported and empowered to be able to care of your child?
- Did you have hospice care for your child at home? What was that experience like?

Bereavement Support:

- After your child died, what was it like for you to try to go back to "normal" life when nothing was normal?
- What were your biggest struggles in those early months?
- Did you feel supported in your grief journey? By your family/friends? Church? Grief support group?
- Did you feel supported by your child's care team? Your child's hospital?
- Are there any ways that you would have liked to receive grief support that didn't happen?
- Did your hospital provide you with resources for grief support?
- If so, what were they?
- Describe the bereavement care you believe a hospital should provide to families after the death of a child.
- Did you have access to or information about grief support groups?
    - Were you given information about grief support groups specific to child loss?
- Would it have helped you to talk with another parent whose child also died?
- If you have other children, how have they been supported in their grief?
- What are your thoughts about providing grief support to your child's care team?
- What are your thoughts about providing grief support to other bereaved parents?
